# Fine Mapping Analysis of the MHC Region to Identify Variants Associated With Chinese Vitiligo and SLE and Association Across These Diseases

**DOI:** 10.3389/fimmu.2021.758652

**Published:** 2022-01-10

**Authors:** Lu Cao, Ruixue Zhang, Yirui Wang, Xia Hu, Liang Yong, Bao Li, Huiyao Ge, Weiwei Chen, Qi Zhen, Yafen Yu, Yiwen Mao, Zhuo Li, Wencheng Fan, Liangdan Sun

**Affiliations:** ^1^ Department of Dermatology, The First Affiliated Hospital of Anhui Medical University, Hefei, China; ^2^ Institute of Dermatology, Anhui Medical University, Hefei, China; ^3^ Key Laboratory of Dermatology, Anhui Medical University, Ministry of Education, Hefei, China; ^4^ Inflammation and Immune Mediated Diseases Laboratory of Anhui Province, Anhui Medical University, Hefei, China; ^5^ Anhui Provincial Institute of Translational Medicine, Anhui Medical University, Hefei, China; ^6^ The Comprehensive Lab, College of Basic Medicine, Anhui Medical University, Hefei, China

**Keywords:** vitiligo, systemic lupus erythematosus, imputation, MHC, fine mapping

## Abstract

The important role of MHC in the pathogenesis of vitiligo and SLE has been confirmed in various populations. To map the most significant MHC variants associated with the risk of vitiligo and SLE, we conducted fine mapping analysis using 1117 vitiligo cases, 1046 SLE cases and 1693 healthy control subjects in the Han-MHC reference panel and 1000 Genomes Project phase 3. rs113465897 (P=1.03×10^-13^, OR=1.64, 95%CI =1.44–1.87) and rs3129898 (P=4.21×10^-17^, OR=1.93, 95%CI=1.66–2.25) were identified as being most strongly associated with vitiligo and SLE, respectively. Stepwise conditional analysis revealed additional independent signals at rs3130969(p=1.48×10^-7^, OR=0.69, 95%CI=0.60–0.79), HLA-DPB1*03:01 (p=1.07×10^-6^, OR=1.94, 95%CI=1.49–2.53) being linked to vitiligo and HLA-DQB1*0301 (P=4.53×10^-7^, OR=0.62, 95%CI=0.52-0.75) to SLE. Considering that epidemiological studies have confirmed comorbidities of vitiligo and SLE, we used the GCTA tool to analyse the genetic correlation between these two diseases in the HLA region, the correlation coefficient was 0.79 (P=5.99×10^-10^, SE=0.07), confirming their similar genetic backgrounds. Our findings highlight the value of the MHC region in vitiligo and SLE and provide a new perspective for comorbidities among autoimmune diseases.

## Introduction

Vitiligo is an acquired depigmentation dermatosis characterized by well-defined porcelain white hypopigmentation patches on the skin, mucosa and hair. Vitiligo is proven to be associated with dysfunction or decreased melanocytes; although manifestations such as depigmentation are not accompanied by fatal symptoms, they do have a certain impact on the mental state and social interactions of patients (1). Systemic lupus erythematosus (SLE) is an inflammatory connective tissue disease characterized by the presence of a variety of autogenic antibodies in the circulatory system, affecting multiple organs and systems, including the skin, heart, kidney, and nerves (2). Numerous epidemiological studies have shown that vitiligo patients have an increased risk of other autoimmune diseases, including autoimmune thyroid disease, SLE, and Addison’s disease (3–5). Moreover, vitiligo shares many susceptibility sites with other diseases, among which HLA is the main locus (6).

The major histocompatibility complex (MHC) region has important effects on both innate and adaptive immunity and contributes to the pathogenesis of a variety of autoimmune diseases. In recent years, an increasing number of susceptibility loci of vitiligo have been found to be scattered throughout both the MHC I and MHC II regions (7–9). Due to polymorphism and linkage imbalance in the MHC region and the shortcoming that variations identified through GWASs are mostly located in non-coding regions, conventional GWASs have been unable to meet the needs of research with regard to the pathogenesis of complex diseases. With the progress of research (10), imputing existing GWAS data to study the entire HLA region has been realized and widely applied to complex diseases, such as psoriasis (11), Graves’ disease (12), and ankylosing spondylitis (13). In fact, HLA-DRB1, HLA-DQA1 (14), and HLA-A (15) have been documented to be associated with vitiligo in the Caucasian population *via* regulation of HLA gene expression, and our team has reported the association of three novel HLA alleles (HLA-DQB1 *02:02, HLA-DQA1*02:01 and HLA-DPB1*17:01) (16) with vitiligo based on the HAN-MHC reference panel (17).

GWASs of SLE have documented nearly 100 risk loci to date. Because of polymorphisms and population correlations, HLA has always been a focus of research and has an influence on disease occurrence, antigenic levels, and clinical presentation. HLA-DRβ1 amino acid 11, HLA-DQβ1 amino acid 45, HLA-A amino acid 156, and HLA-DPβ1 amino acid 76 are independent risk variants for LN (lupus nephritis) (18). HLA-DR and/or DQ molecules regulate autoreactive T cells to participate in the production of SLE-related autoantibodies (19).

To further explore the role of MHC in vitiligo and SLE in the Han Chinese population, we performed MHC imputation using our previous GWAS data and matched a new control group for fine mapping of susceptibility loci for these two diseases. Considering the possibility of shared genetics between immune-related diseases, we also explored the genome-wide pleiotropy of vitiligo and SLE using GWAS data to estimate the correlation of these two diseases from a genetic perspective.

## Materials and Methods

### Study Subjects and Genotyping

Vitiligo patients and SLE patients were recruited according to the criteria of vitiligo European Task Force (20) and systemic lupus international collaborating clinics 2012 revision, respectively. Cases were diagnosed by at least two experts. The controls were recruited as individuals who did not have histories of autoimmune or systemic disorders. Cases and controls were age and geographic region matching. SNP genotyping data were obtained from our previously published paper, as performed using an Illumina 610-Quad Bead Chip (21, 22). In the present study, which included 1117 vitiligo cases, 1046 SLE cases and 1693 healthy controls, written informed consent was obtained from all participants. Quality control of samples and sites was carried out according to a procedure detailed previously (7).

### HLA Imputation

SNPs, InDels and CNVs of the MHC region were imputed using 1000 Genomes Project phase 3 as the reference database. Moreover, HLA alleles and amino acids(AA) were imputed using the HAN-MHC reference panel, which included 10,689 healthy controls recruited from China and contained 29,948 variants. Publication has proved that the CNVs could be obtained by the imputation method using 1KGP phase 3 dataset, while the HAN reference panel also has been reported to have a good performance in HLA alleles and amino acid imputation (17). Therefore, we used two reference panels in this study to perform HLA imputation. Beagle 4.1 (23) was used to perform the imputation process. Data with call rates ≤ 95% or Hardy-Weinberg test results P ≤1.0×10^-3^ were excluded. Imputation dosage R-squared value of >0.9 for SNP; imputation dosage R-squared value of >0.5 for CNV; imputation dosage R-squared value of >0.7 for HLA amino acid and alleles; MAF (minor allele frequency) > 0.01 were set as post-imputation QC criteria.

### Association and Stepwise Regression Analyses

The logistic regression model of Plink 1.9 (https://www.cog-genomics.org/plink2) was used to conduct association analysis for each disease, which was already corrected by sex. Candidate loci associated with the disease were examined using forward stepwise conditional regression analysis, in which top independently associated loci as a covariate were conditioned in the regression model until no significant loci were found. We set a study-wide significance threshold of P=1.32×10^-6^ and P=1.25×10^-6^ for vitiligo and SLE, respectively, on the basis of Bonferroni correction. When the most significant locus was an SNV, the HLA allele or amino acid or CNV in strong linkage disequilibrium (LD, the variants were annotated in the same gene region) with the gene at the locus was selected for conditional regression analysis; otherwise, the SNV was selected as the covariate for conditional regression analysis.

### Genetic Correlation Analysis of Disease

Genome-wide single-nucleotide polymorphism data in the MHC region were used to calculate the genetic correlation between the two diseases with the Bivariate GREML (24) in the Genome-Wide Complex Trait Analysis (GCTA) tool (25), which is based on a linear mixed model and allows for unbiased estimation of the association of complex diseases. To ensure the accuracy of the results, we only retained the results of the single-tailed test less than 0.05.

## Results

### HLA Imputation and Association Analysis

Using Beagle 4.1, we successfully imputed the SNV, CNV, two–digit and four–digit alleles of HLA genes (HLA-A, HLA-B, HLA-C, HLA-DQA1, HLA-DQB1, HLA-DPA1, HLA-DPB1, HLA-DRB1) and amino acids of 2810 vitiligo subjects (1117 cases, 1693 controls) and 2739 SLE subjects (1,693 controls and 1,046 cases). After stringent quality control for vitiligo GWAS imputation data, 37,921 variants in vitiligo and 40,160 variants in SLE were revealed.

### Association of HLA Variants With Vitiligo Susceptibility

**Figure 1:**
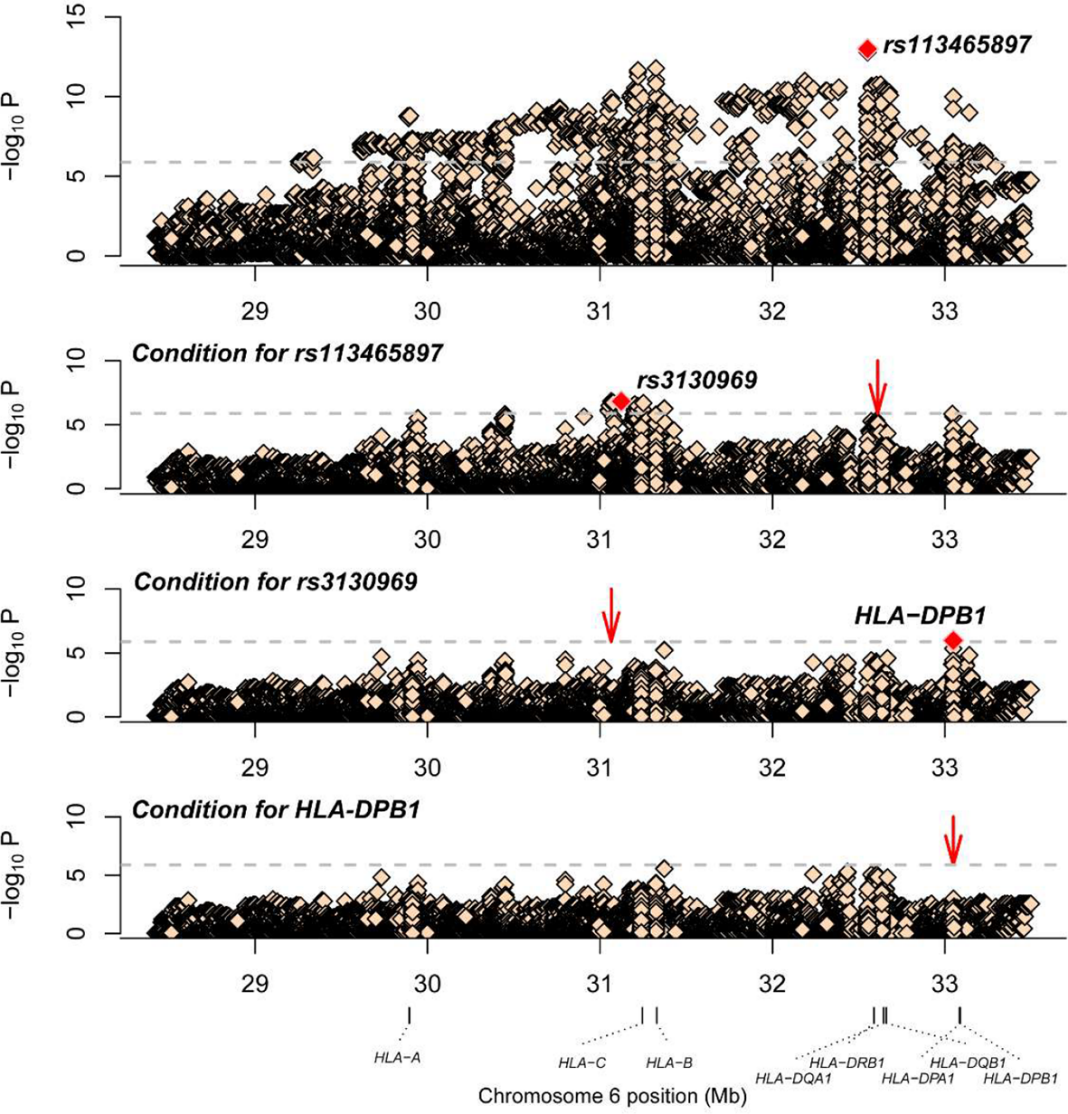
Association plots of stepwise conditional analysis for HLA alleles and SNPs for vitiligo. The abscissa represents genomic loci, and the ordinate represents the -log10(p) of association analysis for each locus. The horizontal line represents the significance threshold P=1.32×10^–6^. The dots marked red in each panel represent the sites used for condition analysis (rs113465897, rs3130969 and HLA-DPB1*0301).

Association studies revealed 1776 variants that reached significance (P< 1.32×10^-6^), including 1725 SNPs, 2 CNVs, 11 HLA alleles (5 two-digit and 6 four-digit) and 38 AA polymorphisms. In the current analysis, the most significant variant was rs113465897 (P= 1.03×10^-13^, OR= 1.64, 95%CI=1.44–1.87), which is located in the HLA-DRB1 gene. The next signal that met the significance threshold while conditioning for rs113465897 was rs3130969 (p=1.48×10^-7^, OR=0.69, 95%CI=0.60–0.79), and conditioning for both SNPs identified a third independently associated signal, HLA-DPB1*0301 (p=1.07×10^-6^, OR=1.94, 95%CI=1.49–2.53) (**Table 1**). There were no significant variants after conditioning for rs113465897, rs3130969 and HLA-DPB1*0301 (**Figure 1**).

**Table 1:** Results of stepwise conditional regression analysis of vitiligo.

STEP	Variant	Variant type	Raw				Stepwise analysis after adjusting		Gene annotation
			Case	Control	OR (95% CI)	P value	OR (95% CI)	P value	
1	rs113465897	SNP	0.30	0.21	1.64 (1.44–1.87)	1.03×10^-13^	N/A	N/A	*Exonic* *HLA-DRB1*
2	rs3130969	SNP	0.19	0.27	0.65 (0.57–0.74)	1.25×10^-10^	0.69 (0.60–0.79)	1.48×10^-7^	*Intergenic* *HCG22, C6orf15*
3	HLA-DPB1*0301	Allele	0.06	0.04	1.86 (1.44–2.39)	1.46×10^-6^	1.94 (1.49–2.53)	1.07×10^-6^	*GeneHLA-DPB1*

N/A, Not Available.

### Association of HLA Variants With SLE Susceptibility

**Figure 2:**
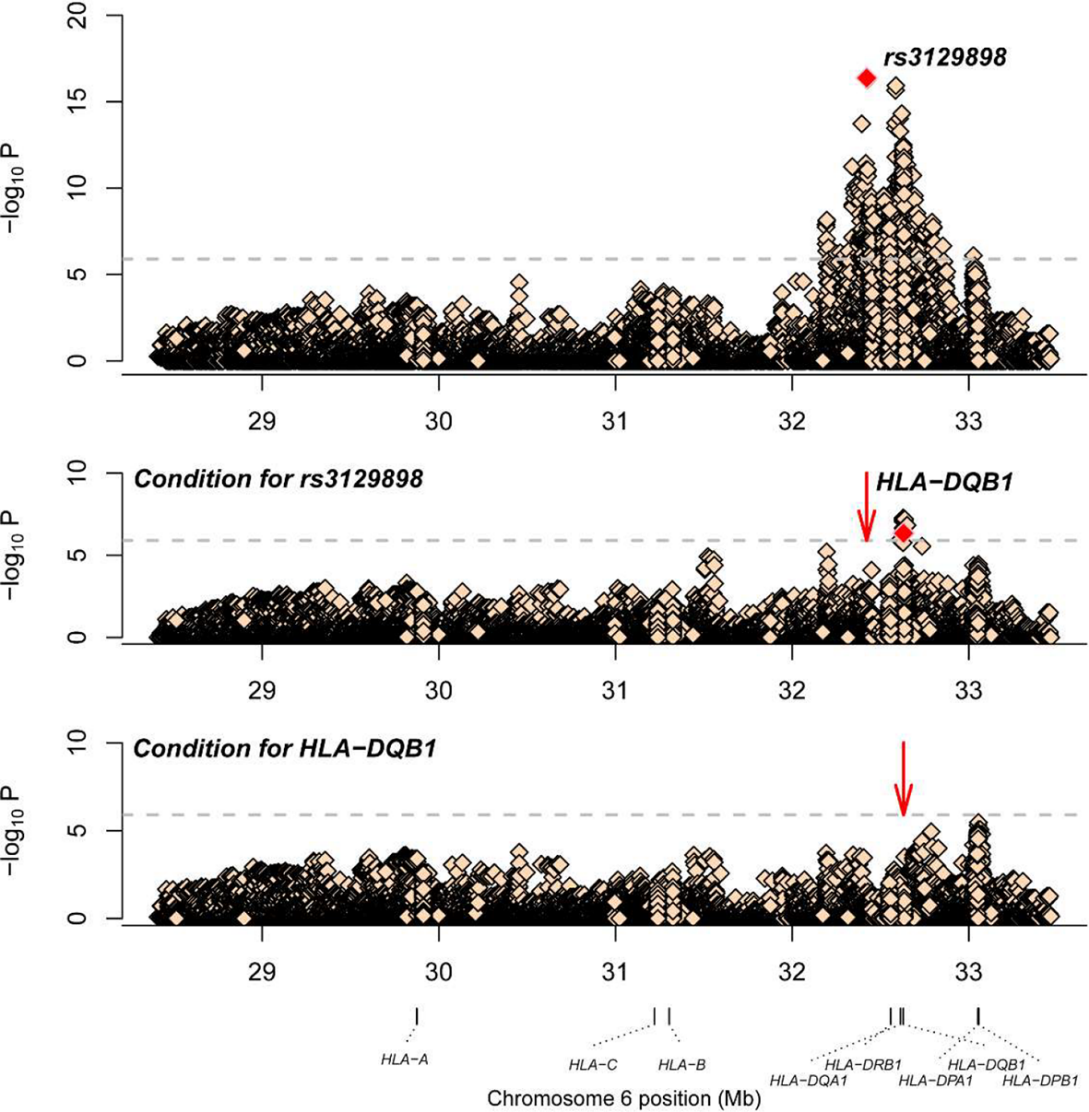
Association plots of stepwise conditional analysis for HLA alleles and SNPs with SLE. The abscissa represents genomic loci, and the ordinate represents the -log10(p) of association analysis for each locus. The horizontal line represents the significance threshold P=1.25×10^–6^. The dots marked red in each panel represent the points used for condition analysis (rs3129898 and HLA-DQB1*0301).

A total of 1266 variants reached significance (P<1.25×10^-6^), including 1217 SNPs, 5 CNVs, 5 HLA alleles (2 two-digit and 3 four-digit) and 39 AA polymorphisms. The most significant locus associated with SLE was rs3129898 (P=4.21×10^-17^, OR=1.93, 95%CI=1.66–2.25), which is located between the HLA-DRA and HLA-DRB5 genes. After conditioning for this site, we defined another remaining significant variant at HLA-DQB1*0301 (P=4.53×10^-7^, OR=0.62, 95%CI=0.52-0.75) (**Table 2**). When conditioning for these two variants, no other signal satisfied the study-wide significance threshold (**Figure 2**). AA_DQB1_140_32629890_A (P=4.52×10^-13^, OR=0.62) and HLA-DQB1*06 (P=2.74×10^-12^, OR=1.66) were the most significant amino acids and alleles, respectively.

**Table 2:** Results of stepwise conditional regression analysis of SLE.

STEP	Variant	Variant type	Raw				Stepwise analysis after adjusting		Gene annotation
			Case	Control	OR (95% CI)	P value	OR (95% CI)	P value	
1	rs3129898	SNP	0.30	0.18	1.93 (1.66–2.25)	4.21×10^-17^	N/A	N/A	*Intergenic* *HLA-DRA,HLA-DRB5*
2	HLA-DQB1*0301	Allele	0.12	0.20	N/A	N/A	0.62 (0.52–0.75)	4.53×10^-7^	*Gene* *HLA-DQB1*

N/A, Not Available.

### Association Between Vitiligo and SLE

The genetic correlation in the MHC region between the two diseases was 0.79 (P=5.99×10^-10^, SE=0.07). Thus, we have strong evidence indicating that genetic factors for vitiligo and SLE correlate positively, especially in the MHC region, and these results were consistent with epidemiological studies.

## Discussion

Researchers have indicated that the pathogenesis of vitiligo is related to genetics, immunity, oxidative stress and other mechanisms, with genetics playing a major role (6). By using the Chinese population-specific reference panel, we found three novel independent association signals (rs113465897, rs3130969 and HLA-DPB1*0301) associated with vitiligo within the MHC region in the Han Chinese population. rs113465897 is a missense variant located in HLA-DRB1 that changes a threonine to an aspartic acid. rs3130969 is located between HCG22 and C6orf15; it is not reported in ClinVar and dpSNP. Several studies have probed the role HLA-DRB1 plays in vitiligo. HLA-DRB1*07 has been reported to be associated with vitiligo, especially early-onset vitiligo in Asian populations (26, 27) and African populations (28). In our present study, HLA-DRB1*0701 was significant (P= 3.01×10^-10^), but significant loci remained when this variant was present. Cavalli et al. reported that higher surface levels of HLA-DR or HLA-DQ bridge the genetic variation in MHC for the development of autoimmunity by enhancing the autoantigen presentation process and increasing the production of cytokines (14). In the Turkish population, the HLA-DRB1*01 allele is protective against vitiligo in terms of oxidative stress; for patients who do not carry the HLA-DRB1*01 allele, the TAC (total antioxidant capacity) level in serum is significantly lower (29). There is no report that vitiligo is associated with either HCG22 or C6orf15, but interestingly, rs9263871 within the class I region (C6orf15-HCG22) has been shown to have the strongest evidence of an association with LN (30). HLA-DPB1*16:01 (31) and HLA-DPB1*17:01 (16) have also been reported to be associated with vitiligo, but HLA-DPB1*03:01 was identified in our study.

Previous studies have shown that MHC plays a crucial role in the etiopathogenesis of SLE, such as triggering the multiplication of T cells, producing antibodies such as anti-Sm, anti-nRPN and anti-DNA, and encoding complement components. We performed MHC fine mapping of SLE risk in the Chinese population and identified rs3129898 and HLA-DQB1*0301 as independent association signals. The significant locus associated with SLE reported by Sun et al. is HLA-DQB1 amino acid position 87 (32), which was also significant in this study (P= 4.63×10^-11^), but rs3129898 was still significant (P= 2.19×10^-08^) after conditioning for HLA-DQB1 amino acid position87. rs3129898 is located between the HLA-DRA and HLA-DRB5 genes, and both have been documented as being associated with the risk of SLE. According to Louthrenoo et al., HLA-DRB5*01:01 is associated with SLE in the Thai population, with a genetic contribution partly related to linkage disequilibrium with DRB1*16:02 (33). HLA-DRA was first found to be associated with SLE in European populations (34), which was later shown in Asian populations (35). HLA-DQB1*0301 has been reported to be associated with SLE susceptibility in Chinese populations (32), as well as in Malays (36) and European (37). In our study, the frequency of the HLA-DQB1*0301 allele was 0.12 in the SLE group and 0.20 in the control group. According to data obtained from Allele Frequency Net Database (http://www.allelefrequencies.net/default.asp), HLA-DQB1*0301 allele frequencies among populations are diverse, with a common frequency in the Chinese population of 0.21; however, this allele has lower frequencies in Italy (frequency = 0.033) and Japan (frequency = 0.112), with ethnicity-specific disparity.

Immunosuppressive therapies are effective for vitiligo patients, and effect on the inner ear and eyes during the progression of the disease indicates the important role of autoimmunity in its pathogenesis. Epidemiology shows that the incidence of comorbid autoimmune disease in patients with vitiligo is significantly higher than that in the general population. Furthermore, even first-degree relatives of patients with vitiligo have an increased prevalence of the same autoimmune disease (38). In general, the prevalence of comorbid autoimmune diseases in vitiligo vary among ethnic groups. A European study found autoimmune comorbidities in 15.4–41.5% of cases (39). Several studies based on different populations prove these data, such as 19–30% in North America (4), 20.3% in Japan (40), and 14.4% in Taiwan (5). The lowest and highest prevalence rates presented in India and Turkey, respectively, are 2.94% (41) and 55% (42). Small sample sizes and consanguineous marriages may contribute to the high comorbidity in Turkey. Both vitiligo and SLE are autoimmune diseases, and they may share a similar genetic background. The frequency of comorbid SLE in vitiligo patients varies among studies, with 0.3% in Michigan, 2.2% in Boston, 0.28% in Taiwan, and 0.47% in New York (4, 5, 43, 44). Research shows that black patients have the highest rate of SLE, at 3%, followed closely by East Asian patients at 2.2% (43). The data vary due to the uneven distribution of ethnic groups and different sample sizes, but when the information is compared with the general population, a statistically higher prevalence is revealed, which is consistent with our findings. Our study showed that the genetic correlation of the MHC region between the two diseases was 0.79, demonstrating not only genetic correlations between vitiligo and SLE but also the substantial role MHC plays in genetic predisposition. Although previous studies have revealed a higher level of antinuclear antibodies (ANAs) in vitiligo patients (45), more than 95% of patients with SLE have a positive ANA test. Due to their high sensitivity but low specificity, ANAs are not directly related to the comorbidities of SLE. The exact cause-and-effect relationship between vitiligo and SLE remains unknown. The overlap in these two diseases and how they function in the occurrence and development of diseases needs to be further explored.

## Conclusion

It is necessary to identify SNPs or variants within the HLA region associated with disease to draw a better conclusion about how to predict disease and to refine the clinical manifestations that may result. In this study, we identified new susceptibility signals associated with risks of vitiligo (rs113465897, rs3130969, HLA-DPB1*0301) and SLE (rs3129898) and confirmed a reported allele, HLA-DQB1*0301, to be associated with SLE. Furthermore, we revealed new genetic predispositions for vitiligo and SLE, advancing research into the functional mechanisms of the disease and demonstrating the correlation of vitiligo and SLE from a population genetics perspective.

## Data Availability Statement

The datasets presented in this study can be found in online repositories. The names of the repository/repositories and accession number(s) can be found below: Zenodo with accession 5768763 - (https://doi.org/10.5281/zenodo.5768763).

## Ethics Statement

The studies involving human participants were reviewed and approved by the Clinical Research Ethics Committee of Anhui Medical University. The patients/participants provided their written informed consent to participate in this study.

## Author Contributions

LS designed the study. QZ, HG, YM, WC, LY, BL, and YY performed the data analysis. XH, ZL, and WF collected the clinical information. LC, RZ, and YW wrote the manuscript and helped with the statistical analysis. All authors contributed to the study and approved the final manuscript.

## Funding

The Research Fund of Anhui Institute of Translational Medicine (ZHYX2020A005), The University Synergy Innovation Program of Anhui Province (GXXT-2020–064) and Clinical Medicine Discipline Construction Project of Anhui Medical University (2021lcxk008) provided support throughout the whole process of the study design, clinical data collection and reagent procurement.

## Conflict of Interest

The authors declare that the research was conducted in the absence of any commercial or financial relationships that could be construed as a potential conflict of interest.

## Publisher’s Note

All claims expressed in this article are solely those of the authors and do not necessarily represent those of their affiliated organizations, or those of the publisher, the editors and the reviewers. Any product that may be evaluated in this article, or claim that may be made by its manufacturer, is not guaranteed or endorsed by the publisher.
